# Chloroquine and Hydroxychloroquine: Efficacy in the Treatment of the COVID-19

**DOI:** 10.3390/pathogens10020217

**Published:** 2021-02-17

**Authors:** Tzu-Chuan Ho, Yung-Hsuan Wang, Yi-Ling Chen, Wan-Chi Tsai, Che-Hsin Lee, Kuo-Pin Chuang, Yi-Ming Arthur Chen, Cheng-Hui Yuan, Sheng-Yow Ho, Ming-Hui Yang, Yu-Chang Tyan

**Affiliations:** 1 Department of Medical Imaging and Radiological Sciences, Kaohsiung Medical University, Kaohsiung 807, Taiwan; r090340@kmu.edu.tw; 2 St. Dominic Catholic High School, Kaohsiung 802, Taiwan; 760176@kmuh.org.tw; 3 Department of Nuclear Medicine, Kaohsiung Medical University Hospital, Kaohsiung 807, Taiwan; chenyi@kmu.edu.tw; 4 Department of Medical Laboratory Science and Biotechnology, Kaohsiung Medical University, Kaohsiung 807, Taiwan; wanchi@kmu.edu.tw; 5 Department of Biological Science, National Sun Yat-sen University, Kaohsiung 804, Taiwan; chlee@mail.nsysu.edu.tw; 6 Graduate Institute of Animal Vaccine Technology, College of Veterinary Medicine, National Pingtung University of Science and Technology, Pingtung 912, Taiwan; kpchuang@g4e.npust.edu.tw; 7 Graduate Institute of Biomedical and Pharmaceutical Science, Fu Jen Catholic University, New Taipei City 242, Taiwan; arthur@kmu.edu.tw; 8 Mass Spectrometry Laboratory, Department of Chemistry, National University of Singapore, Singapore 119077, Singapore; chmyuch@nus.edu.sg; 9 Department of Radiation Oncology, Chi Mei Medical Center, Graduate Institute of Medical Science, Chang Jung Christian University, Tainan 710, Taiwan; shengho@seed.net.tw; 10 Department of Medical Education and Research, Kaohsiung Veterans General Hospital, Kaohsiung 813, Taiwan; 11 Neuroscience Research Center, Graduate Institute of Medicine, College of Medicine, Center for Cancer Research, Research Center for Environmental Medicine, Kaohsiung Medical University, Kaohsiung 807, Taiwan

**Keywords:** coronavirus disease, chloroquine, hydroxychloroquine

## Abstract

Chloroquine (CQ) and its derivative, hydroxychloroquine (HCQ), have attracted wide attention for treating coronavirus disease 2019 (COVID-19). However, conflicting outcomes have been found in COVID-19 clinical trials after treatment with CQ or HCQ. To date, it remains uncertain whether CQ and HCQ are beneficial antiviral drugs for combating COVID-19. We performed a systematic review to depict the efficacy of CQ or HCQ for the treatment of COVID-19. The guidelines of PRISMA were used to conduct this systematic review. We searched through articles from PubMed, Web of Science and other sources that were published from 1 January 2020 to 31 October 2020. The search terms included combinations of human COVID-19, CQ, and HCQ. Eleven qualitative articles comprising of four clinical trials and seven observation studies were utilized in our systematic review. The analysis shows that CQ and HCQ do not have efficacy in treatment of patients with severe COVID-19. In addition, CQ and HCQ have caused life-threatening adverse reactions which included cardiac arrest, electrocardiogram modification, and QTc prolongation, particularly during the treatment of patients with severe COVID-19. Our systematic review suggested that CQ and HCQ are not beneficial antiviral drugs for curing patients with severe COVID-19. The treatment effect of CQ and HCQ is not only null but also causes serious side effects, which may cause potential cardiotoxicity in severe COVID-19 patients.

## 1. Introduction

Coronavirus is a positive-sense, single-stranded RNA virus, including four genera: the alpha, beta, gamma, and delta [1]. It named because of its morphology with crown-like spikes under the electron microscope [1]. Only alpha- and beta-coronavirus can infect humans, and both result in mild or severe respiratory infectious diseases [2,3,4]. In December 2019, the novel coronavirus pandemic began in the city of Wuhan, China and triggered severe acute respiratory syndrome [5]. After that date, WHO declared this disease to be coronavirus disease 2019 (COVID-19) [6]. The etiology of COVID-19 was determined to be severe acute respiratory syndrome coronavirus 2 (SARS-CoV-2, previously known as 2019-nCoV) by the International Virus Nomenclature Committee [6]. SARS-CoV-2 is a beta-coronavirus and shares similar genetic backgrounds with both SARS-CoV (70% similarity), and the Middle East respiratory syndrome MERS-CoV (40% similarity) that previously led to a pandemic coronavirus disease [7]. As of 4 February 2021, SARS-CoV-2 has spread to 223 countries, areas, or territories, and caused 103,362,039 confirmed cases with 2,244,713 deaths globally [8]. It urgently requires effective vaccines and medicines to prevent or treat COVID-19.

**Figure 1:**
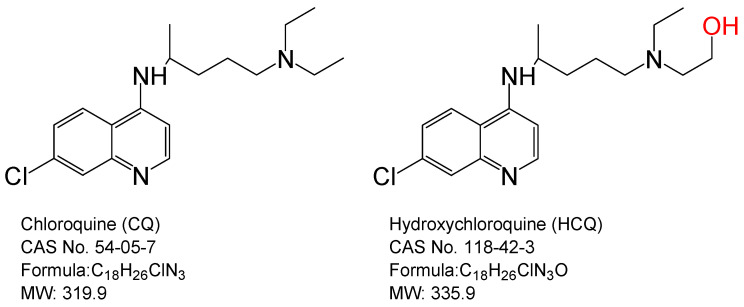
Chemical structures of Chloroquine (CQ) and Hydroxychloroquine (HCQ).

Chloroquine (CQ) and its derivative, hydroxychloroquine (HCQ), are medications used in treatment and prophylaxis of the malaria and autoimmune diseases such as rheumatoid arthritis (RA) and systemic lupus erythematosus (SLE) [9]. The only chemical structure difference between HCQ and CQ is that HCQ has a hydroxyl substitution at the ethyl group of amine (Figure 1). Evidence shows that CQ and HCQ inhibit the infection and replication of SARS-CoV-2 in vitro studies [10,11,12]. CQ and HCQ may have anti-SARS-CoV activity through entry prevention, replication impairment, or immune modulation [13]. CQ and HCQ have become candidates for treatment of COVID-19 due to their anti-SARS-CoV-2 properties and safety for treatment of malaria and autoimmune disease [14]. Clinical findings from Gautret [15] and Gao et al. [16] show that CQ and HCQ contribute to short-term recovery, negative virus conversion, improvement of lung imaging, and inhibition of the exacerbation of pneumonia for hospitalized COVID-19 patients. CQ and HCQ are considered as effective medications to cure COVID-19. However, conflicting results are found in clinical trials of COVID-19 with CQ or HCQ treatment. Studies by Tang [17], Mahevas [18], or Maganoli et al. [19] show that CQ and HCQ both have no clinical efficacy on treatment of COVID-19. To date, it remains uncertain whether CQ and HCQ are beneficial antiviral drugs for combating COVID-19.

The aim of this systematic review was to synthesize articles regarding the cure of COVID-19 by CQ or HCQ and depict the efficacy of these drugs for COVID-19 treatment.

## 2. Method

**Figure 2:**
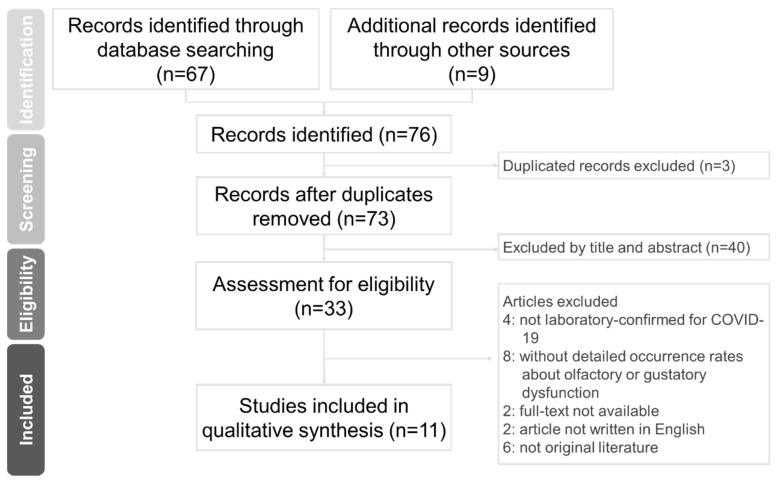
PRIMSA flowchart of study selection.

The guidelines of PRISMA were used to conduct this systematic review (Figure 2). We searched for articles in the PubMed, Web of Science, and other sources that were published from 1 January to 31 October 2020. Searched combination terms included human COVID-19, CQ, and HCQ. This review was focused on using CQ and HCQ in the clinical studies of COVID-19. Publications and preprints were included in this review. The first and second authors were responsible for removing duplicated articles and screened the titles and abstracts. We removed the irrelevant articles after screening. All authors assessed the relevant articles and excluded those without the full-text available, that were not written in English, and that were not original literature. We also excluded clinical studies without detailed occurrence rates about olfactory or gustatory dysfunction or that had subjects which were not laboratory-confirmed to have COVID-19.

## 3. Results

We summarized the results of the search and article selection process in a PRISMA flowchart (Figure 2). Among 76 potentially relevant citations, we finally included 11 studies (four clinical trials and seven observation studies).

Table 1 listed the studies on CQ and HCQ in COVID-19 treatment. There were four studies to test the effect of CQ or HCQ in virus elimination. A study from China showed that CQ can promote the rate of negative virus conversion [16]. By contrast, an open-label randomized clinical trial from China showed that there is no significant difference in the rate of negative virus conversion when using the HCQ treatment plus the standard of cure (SOC) for COVID-19 [17]. The SOC in this study contained therapy with other antiviral drugs. The limitation in this study was that it did not exclude the effect of other antiviral drugs for analysis. In addition, this study also found that the rate of adverse reaction is higher for the HCQ plus SOC group than for the SOC group (30% vs 9%). The adverse reactions mostly were diarrhea in this study. An open-label non-randomized clinical trial showed that the SARS-CoV-2 clearance rate at day 6 after treatment (compared to a self-parallel sample at day 6 before), can achieve 100% in HCQ plus azithromycin (AZ) group, 57.1% in the HCQ alone group, and 12.5% in the control group, respectively [20]. Although HCQ alone can significantly eliminate the SARS-CoV-2, this study suggested that a combination of HCQ and AZ is more effective than HCQ alone for COVID-19 treatment. This study had a limitation because of the number of included participants was significantly smaller for some of the groups. The number of included participants in the groups was 16 in the control group, 14 in HCQ alone, and six in the HCQ plus AZ group. A pilot clinical trial from France [21] also included 11 participants who were more severely ill than the ones in the previous study [20]. It showed only a 20% SARS-CoV-2 clearance rate for days five to six for the HCQ treatment group. This study suggested that HCQ is not effective at eliminating SARS-CoV-2 for patients with severe cases of COVID-19.

There were two studies that evaluated the disease outcome of COVID-19 patients after CQ or HCQ treatment. An observational study from China declared that CQ can improve lung imaging findings and inhibits the exacerbation of pneumonia in patients with mild or moderate COVID-19 cases [16]. This study has some limitations, including unclear intervention and an unknown number of patients in both the control and experimental groups. Similar results were found in a randomized clinical trial with a small sample size from China by using HCQ for patients with mild or moderate cases of COVID-19 [22]. These results were also found in another observational study from France, which was conducted by the previous research team in the above study [20]. We further confirmed the efficacy of a combination treatment of HCQ and AZ for COVID-19 patients. Results showed that 81.3% of COVID-19 patients without underlying disease have a favorable outcome and a shortened discharge time after a combination treatment of HCQ and AZ [15]. However, this is an uncontrolled, non-comparative, observational study with a small sample size.

The effect of HCQ on the mortality rate of treated COVID-19 patients was tested in four observation studies from Italy [24], Belgium [25], the USA [19] and France [18], respectively. Results from observation studies from Italy and Belgium showed that the mortality rate is significantly lower in the HCQ group [24,25]. Studies from the USA and France showed that there is no significant difference in mortality rate between those with or without HCQ [18,19]. In addition, the evidence of these studies showed that the HCQ treated patients have life-threatening adverse reactions such as cardiac arrest [19] and electrocardiogram modification [18]. However, most patients in these studies had severe COVID-19 and some also had underlying diseases [18,19].

Cardiac arrest, electrocardiogram modification and QTc prolongation, which are some of the adverse reactions leading to cardiac death, were also found in another two studies (a double-blinded clinical trial from Brazil [23] and a pilot clinical trial from France without a control group [21]). Most participants in these studies were severely affected COVID-19 patients who had a lower level of consciousness and shortness of breath, and even received nasal oxygen therapy. Of these, the double-blinded clinical trial was terminated early because the high dosage CQ resulted in a high rate of fatality. But the study from Brazil first showed that a high dosage CQ results in toxicity, a red flag, in patients with severe COVID-19.

Overall, there is low confidence in the findings on the efficacy of CQ and HCQ for treatment of patients with mild or moderate COVID-19 due to methodological limitations of the studies such as no control group, unknown intervention and small sample size. These findings showed that CQ and HCQ are not effective for treating patients with severe COVID-19. In addition, CQ or HCQ can cause life-threatening adverse reactions, such as cardiac arrest, electrocardiogram modification and QTc prolongation, particularly during treatment of patients with severe COVID-19.

## 4. Discussion

This systematic review aimed to synthesize the literature regarding the cure of COVID-19 by using CQ and HCQ, and to report the efficacy of CQ and HCQ in the treatment of COVID-19.

Previous studies indicated that CQ and HCQ are anti-malaria drugs, which may increase the endosomal pH and inhibit viruses that rely on low pH to infect cells. For the treatment of COVID-19, indeed, CQ and HCQ may inhibit the spread of SARS-CoV-2 in the African green monkey kidney-derived cell-line Vero, but did not efficiently inhibit SARS-CoV-2 infection of Calu-3 lung cells. This is due to the fact that CQ and HCQ do not appreciably interfere with viral entry nor with the subsequent steps of the viral replication cycle [26]. Actually, some clinical reports also indicated that CQ and HCQ have no efficacy in treatment for patients with severe COVID-19, even resulting in life-threatening side effects for this population. The new evidence of this review suggests that CQ and HCQ are not beneficial antiviral drugs for severe COVID-19 patients.

The major side effects of CQ and HCQ are gastrointestinal upset (vomiting, diarrhea, stomach cramps), skin rash, headache, dizziness and ocular toxicity in patients with malaria or autoimmune disease [27]. These drugs also rarely cause serious side effects including arrhythmia, bronchospasm, angioedema and seizures [27]. In contrast, the serious side effects in severe COVID-19 patients with HCQ or CQ treatment. However, we do not know the mechanism of cardiotoxicity in severe COVID-19 patients by HCQ or CQ. The double-blinded clinical trial from Brazil suggested that the dosage of the drug may be a factor involved in CQ cardiotoxicity [15].

Around 80% of SARS-COV-2 infected individuals have mild or moderate COVID-19 symptoms [28,29]. Hence, these antiviral drugs have the ability to improve the symptoms or eliminate mild or moderate forms of COVID-19, which may in turn contribute to reducing the rate of mortality or viral transmission. Because of methodological limitations, we will require more additional trials to evaluate the efficacy of CQ and HCQ on the treatment of patients with mild or moderate COVID-19.

## 5. Conclusions

This review was hindered by the rapidly increased incidences of COVID-19. As a result there are possible associated research options to be considered for the future. In conclusion, some reports have indicated that CQ and HCQ may inhibit the infection of SARS-COV-2 in Vero cells by blocking virus entry and replication [10,11,12]. Several clinical studies in this systematic review showed that CQ and HCQ may have good effects on promoting the rate of negative virus conversion, eliminating the viral load of SARS-COV-2, improving the lung imaging findings, inhibiting the exacerbation of pneumonia in patients and reducing the discharge time for treatment of patients with mild or moderate COVID-19 [15,16,17,20,22]. However, those results were found under methodological limitations such as not having a control group, unknown interventions and small sample sizes. We will require more clinical evidence to demonstrate the efficacy of CQ and HCQ for treating patients with mild or moderate COVID-19. The risk of cardiotoxicity was found in treated patients with severe COVID-19 [18,19,25]. Severe COVID-19 patients with a high dosage of CQ treatment even had a high rate of fatality, resulting in the early termination of the relevant studies [25]. SARS-COV-2 PCR tests showed that most severe COVID-19 patients had higher positive rates for samples from deep lung [30]. Recently, CQ and HCQ have been found to be unable to block the SARS-COV-2 infection in human lung cells [26], showing that CQ and HCQ have no ability to prevent SARS-CoV-2 from affecting the lungs of severe COVID-19 patients. CQ and HCQ may not effectively treat patients with severe COVID-19 in addition to causing cardiotoxicity and increasing the mortality rate. Current treatment guidelines should be considered cautiously for the use of CQ and HCQ for severe COVID-19 patients.

## Figures and Tables

**Table 1:** Studies on CQ and HCQ in COVID-19 treatment.

Reference	Institution/Country Study Conducted	Design	No. of Participants	Intervention	Results
Gao, J. et al. (2020) [16]	10 hospitals in China in the cities of Wuhan, Jingzhou, Guangzhou, Beijing, Shanghai, Chungging, and Ningbo	Observational study	N = 100 Control group: unlisted Experimental group: unlisted	Unknown	Compared to the control group, CQ improves lung imaging findings, inhibits the exacerbation of pneumonia, and promotes a virus-negative conversion
Magagnoli, J. et al. (2020) [19]	Veterans Health Administration medical centers across the USA	Observational study	N = 807 Control group: no HCQ (n = 395) Experimental group: HCQ alone (n = 198) HCQ + AZ (n = 214)	HCQ alone: 400 mg/daily for 5 days HCQ + AZ: 422.2 mg/daily for 5 days.	Most of participants have chronic disease, such as diabetes and cancer. Compared to the control group, mortality risk is no significantly different in the HCQ group or in the HCQ + AZ group. The HCQ + AZ group has an increased risk of cardiac arrest.
Zhaowei, C et al. (2020) [22]	Hospital of Wuhan University, Wuhan, China	RCT	N = 62 Control group: No HCQ + SOC (n = 31) Experimental group: HCQ + SOC (n = 31)	HCQ, 200 mg, twice daily for 5 days	Severe COVID-19 patients are not enrolled in this study. Compared to the control group, the HCQ group (80.6%, 25/31) have pneumonia improvement and a shorter recovery time for clinical symptoms such as fever and cough. 2 patients in the HCQ group have mild adverse reactions such as rashes and headaches.
Mahevas, M. et al. (2020) [18]	4 French tertiary care centers, France	Observational study	N = 181 Control group: no HCQ (n = 97) Experimental group: HCQ (n = 84)	HCQ, 600 mg/daily for 5 days (starting within 48 h after hospital admission)	The ratios of ICU admission, morality and ARDS development are not significantly different between the no HCQ group and the HCQ group. 8 patients in the HCQ group have electrocardiogram modifications and then HCQ discontinuation.
Gautret, P. et al. (2020) [15]	University Hospital Institute Méditerranée Infection in Marseille, France.	Observation study	N = 80 Control group: Not recruited Experimental group: HCQ + AZ (n = 80, 6 patients from a pervious study)	HCQ, 200 mg thrice daily for 10 days AZ, 500mg/daily for D1 and 250mg/daily for the D2 to D5	81.3% (65/80) of patients have a favorable outcome and are rapidly discharged from the hospital (mean of the discharged day: 4.1 days).
Gautret, P. et al. (2020) [20]	4 centers in Southern France in cities of Marseille, Nice, Avignon and Briançon	Open-label, non-RCT	N = 32 Control group: no HCQ (n = 16) Experimental group: HCQ (n = 20) All group are further classified into three subgroups: asymptomatic, URTI and LRTI.	HCQ, 200 mg, thrice daily for 10 days 6 patients in HCQ group with combination of AZ (500 mg on D1 followed by 250 mg/daily for the D2 to D5) for prevention of bacterial infection	6 days after treatment, the ratio of viral clearance in the HCQ + AZ group, HCQ alone group, and a control group is 100%, 57.1%, and 12.5%, respectively.
Tang, W. et al. (2020) [17]	Ruijin Hospital in Shanghai, China	Open label, RCT, Multicenter	N = 150 Control group: no HCQ + SOC (n = 80) Experimental group: HCQ + SOC (n = 70)	HCQ, 1200 mg/daily on D1 to D3 followed by 800 mg/daily for 2 to 3 weeks SOC, treatment includes another antiviral drug such as arbidol, virazole, lopinavir-ritonavir, oseltamivir, entecavir	98.6% (148/150) of patients have mild or moderate COVID-19 cases. Comparted to the control group, the rate of negative virus conversion is not significantly different in the HCQ + SOC group. The rate of adverse reaction is higher in the HCQ group than that in the control group (30% v.s. 9%).
Borba, MGS.et al. (2020) [23]	Fundação de Medicina Tropical Dr. Heitor Vieira Dourado, Manaus, Amazonas, Brazil	Double-blinded, phase IIb clinical trial	N = 440 (finally enrolled 81 patients for the study) Control group: no CQ from other countries Experimental group: High dosage CQ (n = 41) Low dosage CQ (n = 40)	High dosage CQ, 600 mg twice daily for 10 days Low dosage CQ, 450 mg twice daily on D1 and the 450mg/daily for remaining 4 days.	A high dosage of CQ for 10 days presented toxicity red flags, particularly affecting QTc prolongation. This study was terminated early because of the high dosage CQ resulted in a high rate of fatality.
Molina, J. M. et al. (2020) [21]	Infectious Diseases Department, AP–HP-Saint-Louis Hospital, Paris, France	Polit clinical trial	N = 11 Control group: Not recruited Experimental group: HCQ + AZ	HCQ, 200 mg thrice daily for 10 days; AZ, 500 mg on D1 followed by 250 mg/daily for the D2 to D5	One patient died and another one discontinued treatment due to QTc prolongation. 20% of patients (2/10) have full viral clearance conversion on D6 after treatment.
Castelnuovo, D. A. et al. (2020) [24]	Mediterranea Cardiocentro, Napoli, Italy	Observational study, Multicenter	N = 3451 Control group: no HCQ (n = 817) Experimental group: HCQ (n = 2634)	HCQ, 400 mg twice daily or once daily on D1 and 200 mg/ daily on D2 to D5 or to D10	HCQ treatment results in a 30% lower risk of death in COVID-19 hospitalized patients.
Catteau, L. et al. (2020) [25]	Department of Epidemiology and public health, Sciensano, Brussels, Belgium	Observational study, Multicenter	N = 8075 Control group: no HCQ (n = 3533) Experimental group: HCQ (n = 4542)	HCQ, 2400 mg in total over 5 days	Compared to the control group, the rate of mortality is significantly lower in the HCQ group.

AZ, Azithromycin; RCT, Randomized clinical trial; SOC, Standard of cure; ARDS, Acute respiratory distress syndrome; ICU, Intensive Care Unit; URTI, Upper respiratory tract infection; LRTI, Lower respiratory tract infection.

## Data Availability

The data presented in this study are available on request from the corresponding author.
